# Senescent Vascular Smooth Muscle Cells Drive Inflammation Through an Interleukin-1α–Dependent Senescence-Associated Secretory Phenotype

**DOI:** 10.1161/ATVBAHA.115.305896

**Published:** 2015-08-26

**Authors:** Sarah E. Gardner, Melanie Humphry, Martin R. Bennett, Murray C.H. Clarke

**Affiliations:** From the Department of Medicine, Division of Cardiovascular Medicine, University of Cambridge, Cambridge, United Kingdom.

**Keywords:** aging, atherosclerosis, muscle, smooth, vascular, inflammation, interleukin-1

## Abstract

Vascular smooth muscle cells (VSMCs) that become senescent are both present within atherosclerotic plaques and thought to be important to the disease process. However, senescent VSMCs are generally considered to only contribute through inaction, with failure to proliferate resulting in VSMC- and collagen-poor unstable fibrous caps. Whether senescent VSMCs can actively contribute to atherogenic processes, such as inflammation, is unknown.

**Approach and Results—:**

We find that senescent human VSMCs develop a proinflammatory state known as a senescence-associated secretory phenotype. Senescent human VSMCs release high levels of multiple cytokines and chemokines driven by secreted interleukin-1α acting in an autocrine manner. Consequently, the VSMC senescence-associated secretory phenotype promotes chemotaxis of mononuclear cells in vitro and in vivo. In addition, senescent VSMCs release active matrix metalloproteinase-9, secrete less collagen, upregulate multiple inflammasome components, and prime adjacent endothelial cells and VSMCs to a proadhesive and proinflammatory state. Importantly, maintaining the senescence-associated secretory phenotype places a large metabolic burden on senescent VSMCs, such that they can be selectively killed by inhibiting glucose utilization.

**Conclusions—:**

Senescent VSMCs may actively contribute toward the chronic inflammation associated with atherosclerosis through the interleukin-1α–driven senescence-associated secretory phenotype and the priming of adjacent cells to a proatherosclerotic state. These data also suggest that inhibition of this potentially important source of chronic inflammation in atherosclerosis requires blockade of interleukin-1α and not interleukin-1β.

## Abstract 2

Supplemental Digital Content is available in the text.

Inflammation is an important physiological host defense mechanism that is subverted in multiple chronic diseases. During atherogenesis, it is thought that an innate immune response against factors perceived as pathogens within the vessel wall drives inflammation and initiates plaque growth. Suggested factors include modified lipids, cholesterol and calcium crystals,^1^ and danger signals released from dying and necrotic cells.^2^ These can cause activation of endothelial cells (ECs) and recruitment of leukocytes into the subendothelial space.^3^ Perpetual chronic inflammation results through a poorly understood milieu of cyclic cytokine cascades, activation, recruitment, proliferation and death, and driving atherogenesis. This model is generally supported by mice deficient in the relevant cytokines, receptors, or adhesion molecules, which show modulation of atherosclerosis.^4^ However, much less is known about the role endogenous vessel wall cells play in these processes, and importantly whether they can initiate inflammation.

Most plaques consist of a fibrous cap, composed of vascular smooth muscle cells (VSMCs), collagen, and extracellular matrix that separate a necrotic core from the lumen. In addition, macrophages and T-lymphocytes are present, particularly in the shoulder regions of the plaque. Plaque stability is determined in part by the number of VSMCs and the structural matrix they synthesize.^5^ Myocardial infarction occurs when fibrous cap rupture exposes the underlying necrotic core and matrix, causing thrombosis and vessel occlusion. Many ruptured plaques are clinically silent, suggesting repair and healing commonly occurs. VSMC proliferation and matrix synthesis are crucial to the repair process. However, unstable mature plaques show low levels of VSMC proliferation and clear evidence of VSMC senescence.^6^ Similarly, restenotic neointimas also contain senescent VSMCs.^7^ Senescence is a protective mechanism engaged in response to excessive stress that induces permanent cell cycle arrest to prevent transmission of defects to the next generation, particularly to stop malignant transformation. Senescence can occur after repeated cell division (replicative senescence) or be induced by mitochondrial deterioration, DNA damage or oxidative stress—all processes VSMCs are exposed to within plaques. Because VSMC proliferation and matrix production are required to stabilize the fibrous cap, senescence is suggested to promote plaque instability. Thus, senescent VSMCs are generally considered to be bystanders that only contribute to atherogenesis through inaction.

On the contrary, many senescent tumor cells develop altered secretory activities, known as a senescence-associated secretory phenotype (SASP). SASPs contain proinflammatory cytokines (eg, interleukin-6 [IL-6], IL-1) and chemokines (eg, IL-8, GROα), growth factors (eg, granulocyte-colony stimulating factor [G-CSF], basic fibroblast growth factor [bFGF]), proteases (eg, matrix metalloproteinases [MMPs], plaminogen activator inhibitor-1 [PAI-1]), but never anti-inflammatory factors.^8^ Thus, the mitostatic effect of senescence is counterbalanced by the effects of the SASP on the local milieu. In many cases, the SASP is driven by cell surface IL-1α^9^ and secreted IL-1β,^10^ which both bind the IL-1 receptor type 1 (IL-1R1) and induce identical signaling. IL-1–mediated effects, such as cytokine production, EC/macrophage activation, increased adhesion molecules, and increased vascular permeability^11^ are all important during atherogenesis. Indeed, atherosclerosis in mice is increased with more IL-1 signaling or decreased with less.^12–15^

We find that senescent VSMCs establish a proinflammatory SASP, characterized by chronic secretion of multiple proatherogenic cytokines and active MMP9, but downregulation of collagen. This SASP acts as a potent stimulus for the chemotaxis of mononuclear cells in vitro and in vivo. Senescent VSMCs upregulate multiple inflammasome components with the autocrine secretion of IL-1α solely responsible for driving the SASP. Furthermore, IL-1α also primes adjacent VSMCs and ECs to a proinflammatory and proadhesive state. These data imply that senescent VSMCs may actively drive chronic inflammation and matrix destruction in plaques.

## Materials and Methods

Materials and Methods are available in the online-only Data Supplement.

## Results

### Senescent VSMCs Are a Feature of Advanced Plaques, Which Can Be Modeled In Vitro

**Figure 1.**
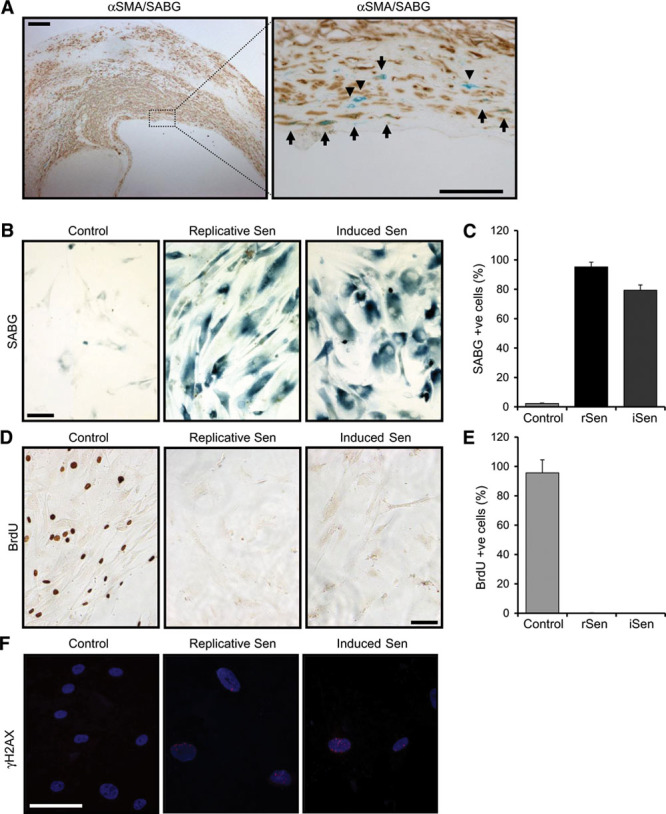
Vascular smooth muscle cell (VSMC) senescence, which occurs in mature plaques, can be modeled in vitro. **A**, Human carotid plaque stained for senescence with senescence-associated β-galactosidase (SABG; blue) and VSMCs with α-smooth muscle actin (αSMA; brown), as indicated. Most SABG +ve cells are in the fibrous cap region, whereas dual staining shows the majority of senescent cells to be VSMCs (arrows), although some senescent cells with a VSMC-like morphology do not stain for αSMA (arrow heads). **B**–**F**, Replicative and induced senescent VSMCs were stained and enumerated for SABG (**B** and **C**) and BrdU (5-bromo-2’-deoxyuridine; **D** and **E**) +ve cells, or stained for persistent DNA damage with γH2AX (**F**). Data represent mean±SD, representative of n=≥3. Scale bars represent 200 mm (low power) and 50 mm (high power).

Aging is the dominant risk factor for atherosclerosis, with senescent cells a key feature of aged tissues in humans. Although there are no completely specific markers for senescent cells, many cell types express a pH-labile enzyme called senescence-associated β-galactosidase (SAβG) when they undergo senescence because of multiple stimuli. Advanced human atherosclerotic plaques expressed SAβG-positive cells in the intima, most marked in the fibrous cap (Figure 1A; Figure I in the online-only Data Supplement). Double labeling revealed ≈70% of these cells express α-smooth muscle actin (Figure 1A), indicative of VSMC origin. In addition, senescent cells are also observed in peripheral regions overlying less mature plaques (Figure I in the online-only Data Supplement).

Senescence can be induced by multiple stimuli, for example, replicative exhaustion, DNA damage, and activation of oncogenes,^8^ and often occurs because of the combined effect of these factors. VSMCs undergo replicative senescence after 12 to 20 population doublings, but this is greatly accelerated by DNA damage.^16^ Thus, we studied both replicative and DNA damage–induced senescence in VSMCs. Human VSMCs derived from disease-free aorta were cultured until senescent or pulsed with the DNA damaging agent bleomycin. Both VSMC cultures showed an enlarged flattened morphology and SAβG activity in ≥80% of cells (Figure 1B and 1C). Bleomycin-treated VSMCs showed both a dose and time-dependent increase in SAβG activity with 50 µg/mL of bleomycin giving ≈80% positive cells by day 11 (Figure II in the online-only Data Supplement), but without the cell death seen with higher doses (Figure III in the online-only Data Supplement). Importantly, both senescent cultures showed <5% proliferation, as evidenced by BrdU (5-bromo-2’-deoxyuridine) incorporation (Figure 1D and 1E; Figure IV in the online-only Data Supplement), and persistent unrepaired DNA damage, as evidenced by increased nuclear foci of γ-H2AX (Figure 1F; Figure V in the online-only Data Supplement), which colocalized with SAβG-positive VSMCs (Figure VI in the online-only Data Supplement). Once established, senescent cells were remarkably stable showing low rates of cell loss (Figure VII in the online-only Data Supplement), with cultures of replicatively senescent cells stable in culture for >180 days (Figure VII in the online-only Data Supplement). This combination of SAβG staining, flattened morphology, low proliferation, and persistent DNA damage are features of senescent cultures in multiple cell types,^8^ confirming our models of VSMC senescence.

### Senescent VSMCs Secrete Multiple Inflammatory Cytokines in an IL-1α–Dependent Manner

**Figure 2.**
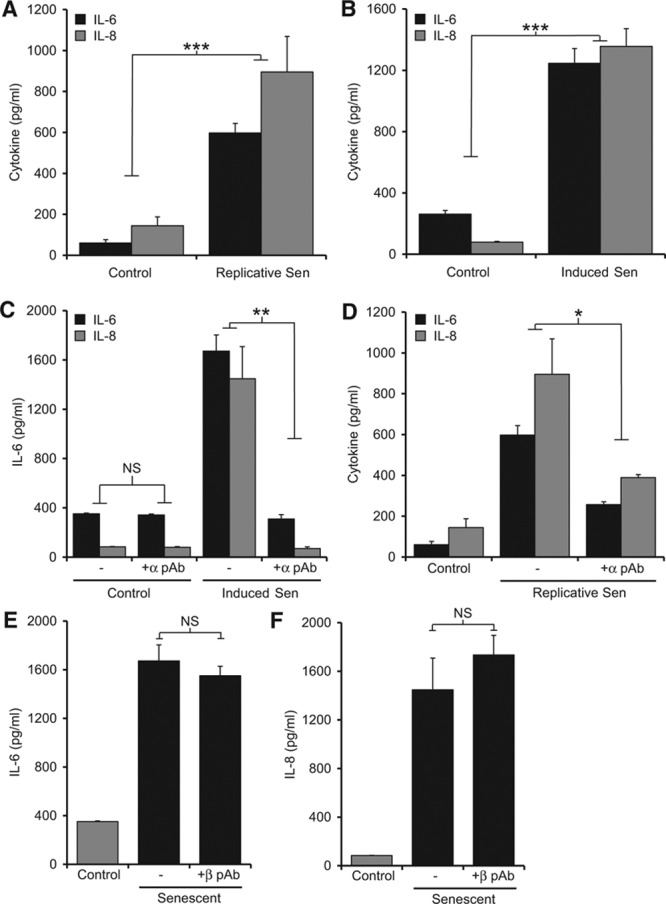
Senescent vascular smooth muscle cells (VSMCs) secrete cytokines and chemokines in an interleukin-1α (IL-1α)–dependent manner. Cytokine and chemokine content of conditioned media from control, replicative (**A**) and induced (**B**) senescent VSMC cultures measured by ELISA. IL-6 and IL-8 content of conditioned media from control and senescent VSMCs incubated ±neutralizing IL-1α (**C** and **D**) or IL-1β antibodies (**E** and **F**). Data represent mean±SEM of n=5 (**A**–**C**, **E**, and **F**), 3 (**D**); *P≤0.05, **P≤0.02, and ***P≤0.005. NS indicates not significant.

A key feature of senescence in many cell types is a SASP, whereby a large range of cytokines and chemokines are persistently secreted.^8^ Human VSMC senescence was associated with secretion of high levels of the classic SASP cytokine IL-6 and chemokine IL-8 in both replicatively senescent (Figure 2A) and induced senescent (Figure 2B) VSMC cultures. Extended profiling by cytometric bead array (Figure VIII in the online-only Data Supplement) and antibody array (Figure IX in the online-only Data Supplement) revealed that senescent VSMCs also secrete increased levels of the powerful leukocyte chemoattractants monocyte chemoattractant protein-1 (MCP-1), macrophage inflammatory protein-1α (MIP-1α)/CCL3, and MIP-1β/CCL4, and reduced levels of RANTES and the IL-1 antagonist soluble IL-1R2. As *IL6*, *IL8*, and *MCP1* are IL-1 responsive genes, we investigated whether IL-1 induces the SASP in senescent VSMCs. Specific blockade of IL-1α prevented the SASP, with significant reduction of both IL-6 and IL-8 in both induced (Figure 2C) and replicatively senescent cultures (Figure 2D), whereas blockade of IL-1β did not (Figure 2E and 2F). Together this suggests that the SASP in senescent VSMC cultures is driven by IL-1α and that induced and replicative senescence share common features and SASP components in VSMCs. As replicative senescence requires long-term culture and cannot provide passage-matched control cells, we investigated specific effects of the SASP factors with induced VSMC senescence.

### Physiological Secretion of IL-1α by Senescent VSMCs Drives the SASP in an Autocrine Manner

**Figure 3.**
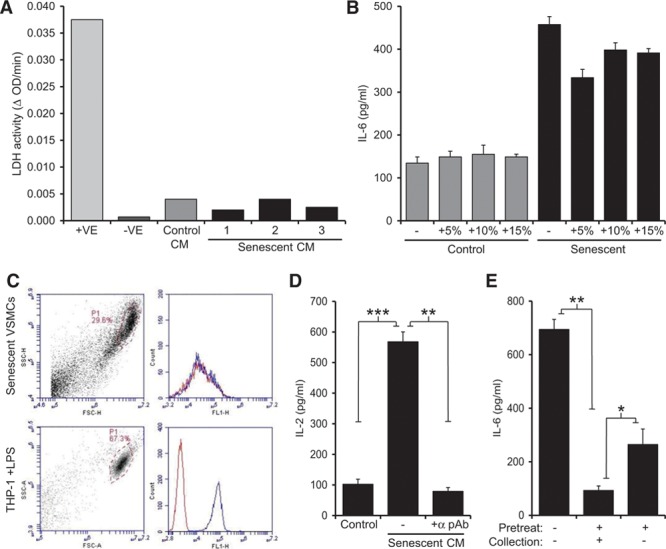
Secretion of interleukin-1α (IL-1α) by senescent vascular smooth muscle cells (VSMCs) drives the senescence-associated secretory phenotype in an autocrine manner. **A**, Lactate dehydrogenase (LDH) activity in conditioned media (CM) from control and senescent VSMCs, lysed cells (+ve) or buffer only (−ve). **B**, IL-6 content of conditioned media from control and senescent VSMCs incubated with the indicated % of necrotic control and senescent VSMCs. **C**, Flow cytometry analysis of senescent VSMCs and lipopolysaccharide (LPS)-treated THP-1 cells stained with isotype control-FITC (fluorescein-5-isothiocyanate; red) or anti-IL-1α−FITC (blue). **D**, IL-1–dependent IL-2 production by EL4 cells incubated with senescent VSMC conditioned media (CM), ±neutralizing IL-1α pAb. **E**, IL-6 content of media conditioned by senescent VSMCs incubated ±IL-1α pAb as a pretreatment or during supernatant collection. Data represent mean±SEM of n=3 (**B** and **E**), 4 (**D**); *P≤0.05, **P≤0.02, and ***P≤0.005.

IL-1α has been varyingly reported to be exclusively cytoplasmic, physiologically secreted by inflammasome-dependent^17,18^ or -independent^19^ mechanisms, and also to exist as a cell membrane–tethered mature form.^20^ In addition, IL-1α is a key danger signal that induces inflammation on release from necrotic cells.^2,21,22^ We therefore determined whether the IL-1α–dependent SASP in senescent VSMC cultures was because of secretion, a membrane-tethered form, or potentially release from necrotic cells. Lactate dehydrogenase as a marker of necrosis was similar in the conditioned media from control and senescent VSMCs (Figure 3A). In addition, the deliberate spiking of control and senescent VSMC cultures with necrotic VSMCs at typically observed levels did not increase IL-6 release (Figure 3B). Together these data imply that the IL-1α–dependent SASP is not because of increased leakage from necrotic cells in senescent VSMC cultures. Previous work that reported a role for IL-1α in the maintenance of SASPs suggested it to be exclusively mediated by cell-surface IL-1α.^9^ We could not find IL-1α on the surface of senescent VSMCs by flow cytometry, in contrast to lipopolysaccharide-activated THP-1 cells used as a positive control (Figure 3C). In contrast, the conditioned media from senescent VSMCs contained active IL-1α, as evidenced by the specific reduction in IL-1–dependent IL-2 production with an IL-1α neutralizing antibody (Figure 3D) and by ELISA (Figure X in the online-only Data Supplement).

We next sought to determine whether IL-1α is a proximal initiator of the VSMC SASP or acts more as a distal effector of the factors released. Senescent VSMCs cultured continuously with IL-1α blockade released basal levels of IL-6 (Figure 3E). However, short-term release (6 hours) of IL-1α blockade resulted in the immediate reestablishment of the SASP, as evidenced by the rapid secretion of IL-6 into the conditioned media (Figure 3E). This suggests that although IL-1α drives the production of SASP factors, its inhibition does not phenotypically alter a senescent cell, implying IL-1α is the apical downstream effector of SASPs rather than an upstream inducer of senescence.

### Senescent VSMCs Induce Chemotaxis, Secrete Active MMP-9, and Upregulate Inflammasome Components

**Figure 4.**
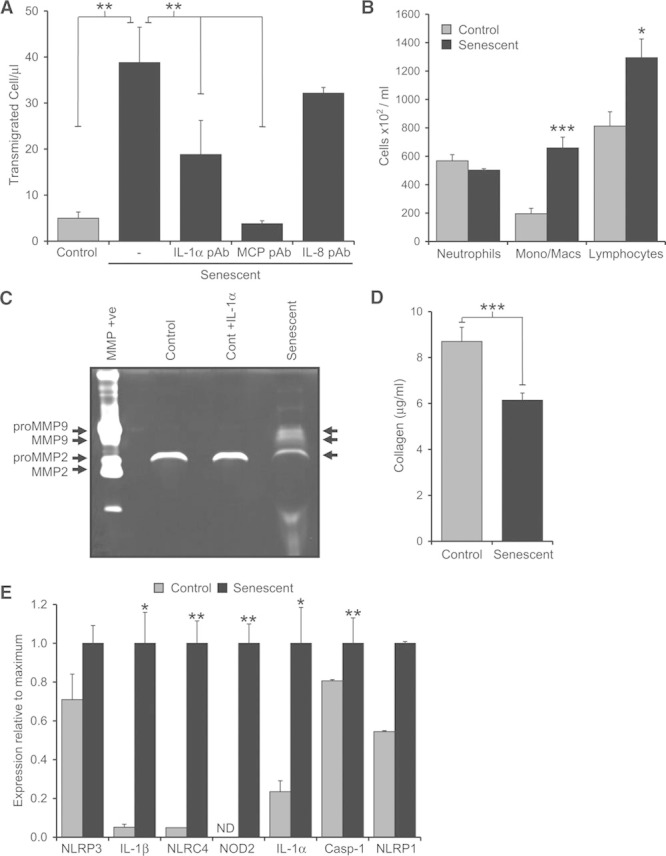
Senescent vascular smooth muscle cells (VSMCs) induce chemotaxis, secrete active matrix metalloproteinase-9 (MMP-9), and upregulate inflammasome components. **A**, Number of THP-1 cells migrating toward control or senescent VSMCs, ±neutralizing interleukin-1α (IL-1α), MCP-1, or IL-8 antibodies. **B**, Enumeration of immune cells recruited to the peritoneum of mice in response to control or senescent VSMCs. **C**, Gelatin gel zymography showing level and activity of MMPs in the conditioned media of control, IL-1α–treated or senescent VSMCs. **D**, Amount of collagen produced by equal numbers of control or senescent VSMCs. **E**, Relative expression of transcripts for inflammasome-associated components in control or senescent VSMCs. Data represent mean±SEM of n=4 (**A**), 3 (**B** and **D**), 2 (**E**); *P≤0.05, **P≤0.02, ***P≤0.005. ND indicates not detected.

Our data suggest that senescent VSMCs actively secrete IL-1α that drives the SASP in an autocrine/paracrine fashion. Furthermore, in contrast to senescent cell types that have surface IL-1α that is likely to only affect neighboring cells, IL-1α secreted from senescent VSMCs might stimulate more distant cells. A key feature and driver of atherogenesis is the continual recruitment of monocytes from the circulation; we therefore investigated whether senescent human VSMCs could induce chemotaxis of mononuclear cells. THP-1 cell migration was increased 8-fold toward senescent than control VSMCs (Figure 4A). Migration was inhibited significantly by treatment of the senescent VSMCs with an IL-1α neutralizing antibody before the assay or an MCP-1 neutralizing antibody added during the chemotaxis assay (Figure 4A). Blocking IL-8 during the assay did not reduce recruitment. This indicates that IL-1α drives production of chemoattractants from senescent VSMCs and that the main chemoattractant of monocytic cells within the SASP is MCP-1. To confirm that senescent VSMCs could also induce leukocyte recruitment in vivo, we injected live VSMCs into the peritoneal cavity of mice. Again, senescent VSMCs caused significantly more recruitment of monocyte/macrophages and lymphocytes, but not neutrophils, compared with control VSMCs (Figure 4B; Figure XI in the online-only Data Supplement).

We also examined the effect of VSMC senescence on other IL-1–induced genes, and the effect of senescence on expression of inflammasome components that maybe required for IL-1α secretion. MMP plays a key role in degrading the structural matrix that strengthens the fibrous cap and are secreted by some senescent tumor cells. Using gelatin zymography, we find that control VSMCs secrete pro-MMP2. In contrast, senescent VSMCs release pro-MMP2, pro-MMP9, and active MMP9 (Figure 4C). IL-1–primed control VSMCs did not secrete active MMP9, suggesting this process to not only be IL-1–dependent in VSMCs. As VSMC-produced structural matrix maintains the fibrous cap, we assessed collagen production and found senescent VSMCs generate significantly less than control cells (Figure 4D). Senescent VSMCs also showed polarization between low and high α-smooth muscle actin expression (Figure XII in the online-only Data Supplement). Secretion of IL-1α has been suggested to require active caspase-1,^17,18^ and thus assembly and activation of inflammasomes. Using quantitative polymerase chain reaction, we profiled control and senescent VSMCs for levels of inflammasome components and associated factors. Senescent VSMCs contained significantly increased levels of transcripts for IL-1β, IL-1α, NLR family, CARD domain containing 4 (NLRC4), nucleotide-binding oligomerization domain-containing protein 2 (NOD2), and NACHT, LRR, and PYD domains-containing protein 1 (NLRP1) (Figure 4E), indicative of a shift toward a more proinflammasome phenotype.

### VSMC SASP Primes Adjacent Control Cells to a Proinflammatory State

**Figure 5.**
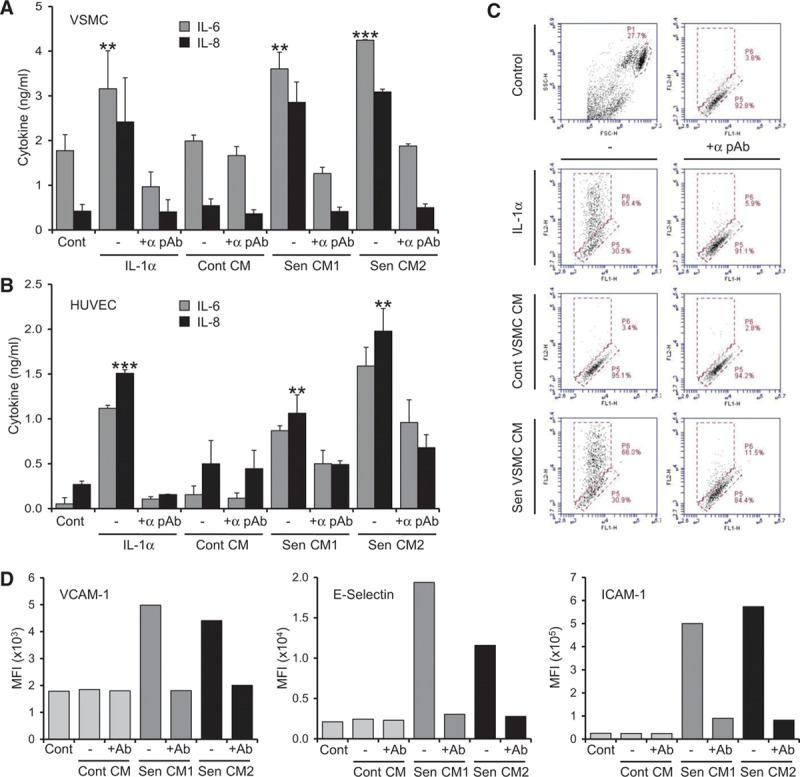
Vascular smooth muscle cell (VSMC) senescence-associated secretory phenotypes (SASPs) can prime adjacent control cells to a proinflammatory state. **A** and **B**, Interleukin-6 (IL-6) and IL-8 content of cell lysates from control VSMCs (**A**) or human umbilical vein endothelial cells (HUVECs; **B**) incubated with IL-1α or conditioned media (CM) from control or 2 senescent VSMC cultures, ±neutralizing IL-1α pAb. IL-8 values are scaled 2.5-fold lower. **C**, Flow cytometry plots showing IL-1α–dependent increase in surface E-selectin expression after treatment as indicated. **D**, Mean fluorescent intensities (MFIs) of adhesion molecules on HUVEC populations analyzed by flow cytometry after treatment as indicated, ±neutralizing IL-1α pAb (Ab). Data represent mean±SD of n=3 (**A**), 2 (**B**–**D**); **P≤0.02 and *** *P*≤0.005.

As senescent VSMCs secrete IL-1α, and the classic inflammasome priming toll-like receptor ligands (eg, lipopolysaccharide) induce equivalent downstream signaling as IL-1, we investigated whether IL-1α could also prime local control VSMCs or macrophages to a proinflammasome phenotype. Treatment of control VSMCs with IL-1α led to an upregulation of inflammasome components comparable with that seen in senescent VSMCs (Figure XIII in the online-only Data Supplement), although macrophages showed no response to IL-1α (not shown). Importantly, given that the SASP is a complex mixture of cytokines, we determined whether conditioned media from senescent VSMCs could activate normal VSMCs and ECs. As this conditioned media already contains SASP factors, we treated target cells with bafilomycin A to block protein secretion, and assayed factors within cell lysates after exposure to conditioned media from control or senescent VSMCs. Conditioned media from senescent VSMCs led to IL-1α–dependent production of IL-6, IL-8 (Figure 5A and 5B), and MCP-1 (Figures XIV and XV in the online-only Data Supplement) in both VSMCs and ECs, whereas media from control VSMCs did not. Furthermore, senescent VSMC conditioned media caused the IL-1α–dependent upregulation of the adhesion molecules vascular cell adhesion molecule 1 (VCAM-1), intercellular adhesion molecule 1 (ICAM-1), and E-selectin in ECs (Figure 5C and 5D). This suggests that in addition to the direct release of inflammatory factors, senescent VSMCs can cause normal cells to release cytokines and upregulate adhesion molecules.

### Senescent VSMCs Have a High Energy Demand That Allows Their Selective Elimination

**Figure 6.**
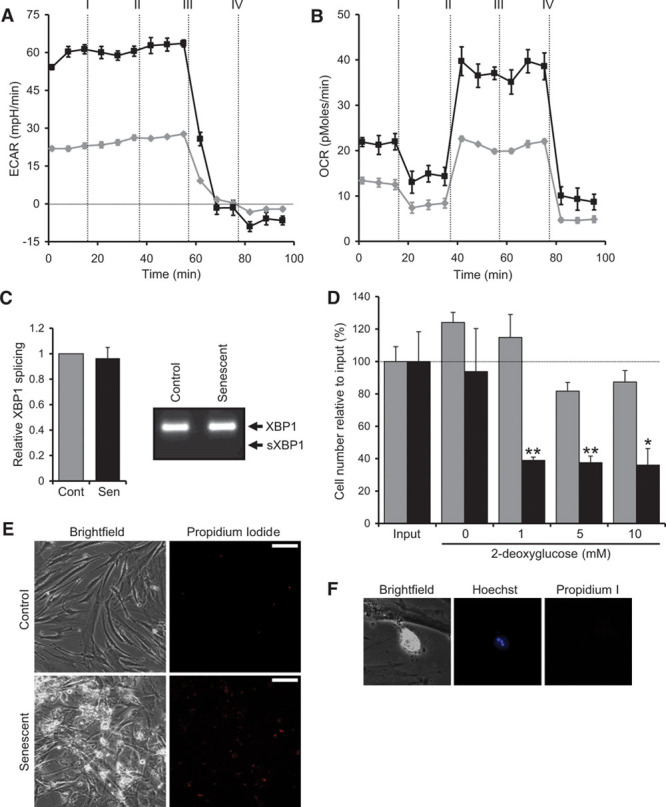
Senescent vascular smooth muscle cells (VSMCs) adopt a hypermetabolic state that enables their selective elimination. **A** and **B**, Measurement of glycolysis by the extracellular acidification rate (ECAR; **A**) and oxidative phosphorylation by the oxygen consumption rate (OCR; **B**) in control (gray) and senescent (black) VSMCs treated at times indicated with: I=oligomycin; II=FCCP; III=2-deoxyglucose (2-DG); and IV=rotenone/antimycin. **C**, Level of XBP-1 splicing in control and senescent VSMCs measured by qPCR (quantitative polymerase chain reaction) and RT-PCR (reverse transcription-polymerase chain reaction). **D**, Cell number measured by crystal violet following treatment of control (gray) and senescent (black) VSMCs with 2-DG for 4 days. **E**, Propidium iodide staining reveals increased levels of cell death in senescent VSMCs after 2-DG treatment. **F**, Senescent VSMC treated with 2-DG displaying cellular blebbing, pyknotic nuclear condensation, and propidium iodide negativity indicative of apoptosis. Data represent mean±SD of n=2; *P≤0.05, **P≤0.02. Scale bars represent 50 mm.

The transcription, translation, processing, and secretion of multiple proteins at high concentration are an intrinsically energy-intensive process. Furthermore, such demand can lead to misfolded proteins triggering the unfolded protein response—another energy consuming process. To test whether the VSMC SASP necessitates metabolic alterations to meet such energy demands, we used a Seahorse extracellular flux analyzer. Measurement of the extracellular acidification rate indicated senescent cells to have glycolytic rates ≈2.6-fold higher than control cells (Figure 6A), which was shown to be specific by abolishment with the glycolytic inhibitor 2-deoxyglucose (Figure 6A). Similarly, the oxygen consumption rate indicated senescent VSMCs to use ≈2-fold more oxidative phosphorylation than control cells (Figure 6B), with a higher basal, maximal, and spare respiratory capacity. However, senescent VSMCs show no active unfolded protein response, as evidenced by a lack of spliced X-box binding protein 1 (XBP-1; Figure 6C). This indicates that senescent VSMCs adapt to a hypermetabolic and hyperbiosynthetic state to enable the high-level protein production of the SASP without proteotoxic stress. Metabolic targeting is a developing potential anticancer therapy that relies on the altered metabolic requirements of tumor cells.^23^ Given the hypermetabolic state of senescent VSMCs, we investigated whether a similar strategy could lead to their selective elimination. Treatment of VSMCs with 2-deoxyglucose (a glucose analog that competitively inhibits glycolysis) lead to significantly more death of senescent than control cells (Figure 6D and 6E). Dying cells showed features of apoptosis, such as pyknotic nuclear condensation and cellular blebbing (Figure 6F). Together, these data suggest that the high energy demand of senescent VSMCs could enable metabolic targeting and potentially their selectively elimination.

### Senescent Cells Within Human Carotid Plaques Express IL-1α and Colocalize With IL-6 and CD68

**Figure 7.**
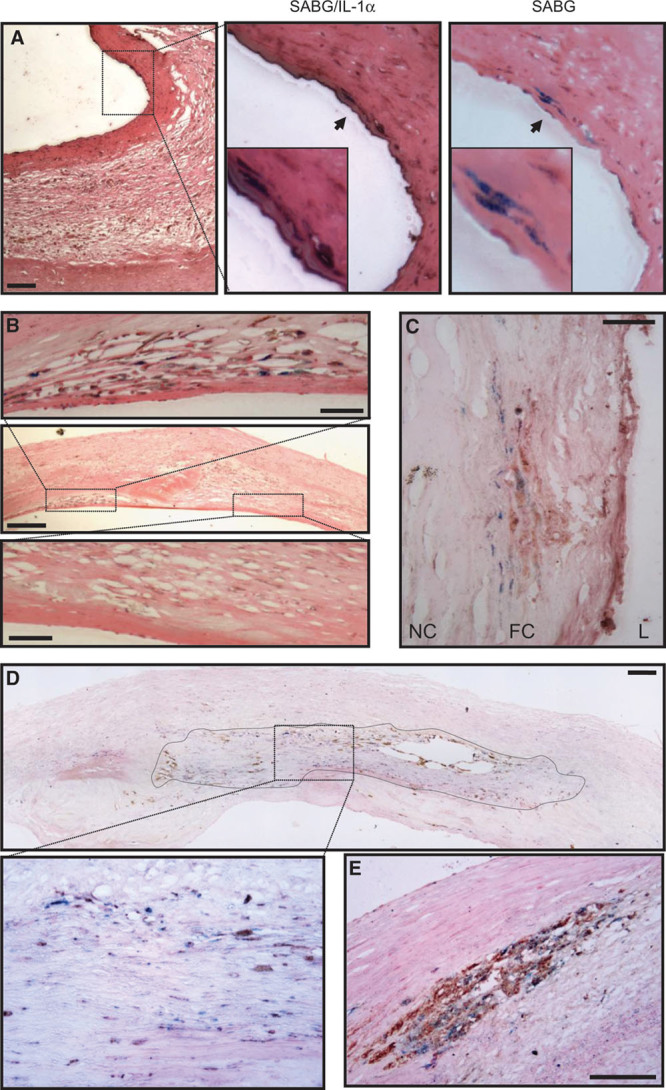
Senescent cells in human carotid plaques express interleukin-1α (IL-1α) and colocalize with IL-6 and CD68. Human carotid endarterectomy samples stained for senescence-associated β-galactosidase (SABG): showing senescent vascular smooth muscle cell (VSMC)-like cells in the fibrous cap that coexpress IL-1α (**A**); a discrete region containing many senescent cells showing localized IL-6 expression, in contrast to an adjacent region with no senescent cells or IL-6 (**B**); area within a fibrous cap (FC) showing high levels of local IL-6 expression adjacent to senescent cells (**C**); a large composite image showing localization between senescent and CD68 +ve cells (outlined and enlarged below; **D**); a region showing focal accumulation of senescent and CD68 +ve cells (**E**). Only eosin counterstain was used. Scale bars represent 100 and 25 mm (**B**; high power). L indicates lumen; and NC, necrotic core.

To assess whether these findings are of significance to atherosclerosis in vivo, we examined SAβG-stained human carotid plaques. Senescent VSMC-like cells within the fibrous cap showed increased expression of IL-1α compared with adjacent SAβG-negative cells (Figure 7A). As we have shown that the VSMC SASP induces cytokine expression in adjacent cells, we examined plaques for regions of SAβG-positive cells that had increased local IL-6 expression. SAβG-positive cells showed both IL-6 expression and increased IL-6 expression in adjacent cells (Figure 7B and 7C), including IL-6 expression in the overlying endothelium (Figure 7C). Given that senescent VSMCs cause chemotaxis of mononuclear cells via MCP-1, we examined whether increased levels of CD68-positive cells were found in the vicinity of SAβG-positive cells. Large areas of the plaque could be seen to have increased levels of CD68-positive and SAβG-positive cells near to one another (Figure 7D), whereas other areas showed a focal accumulation of both SAβG- and CD68-positive cells (Figure 7E). Together, these data suggest that senescent cells in human plaques express IL-1α that can drive increased local expression of inflammatory cytokines, causing attraction of mononuclear cells.

## Discussion

Atherosclerosis is a disease that develops over decades in humans. Although many atherogenic processes and epidemiological risk factors are known, no evidence of causation exists in humans. Most agree that inflammation is a key initiator and driver of the disease, but what initial insult or factors drive this and why it does not resolve is unclear. Senescent VSMCs are found in human plaques^16^ (Figure 1A) and are thought to contribute to the disease. However, they are observed to be rare and therefore how so few cells can have any functionally important effect is unclear. Furthermore, it is not known whether any effect of VSMC senescence is only through inaction, or if they can actively drive atherogenic processes such as inflammation.

We find that senescent human VSMCs may actively contribute to atherogenesis and plaque rupture through the establishment of a SASP. The SASP is characterized by the persistent release of multiple inflammatory cytokines and chemokines, which is driven by autocrine stimulation of senescent VSMCs by IL-1α. These powerful chemotactic signals cause recruitment of monocytes and lymphocytes in vitro and in vivo, whereas secreted IL-1α activates adjacent normal VSMCs and ECs, resulting in the amplification of proinflammatory cytokine release and expression of adhesion receptors. Senescent VSMCs also secrete less collagen and more active MMP9 that can degrade collagen within the structural matrix, which could further weaken the plaque. This persistent high-level expression of secreted proteins necessitates a hypermetabolic state sustained by increased levels of glycolysis and oxidative phosphorylation. This renders senescent VSMCs sensitive to metabolic targeting with glycolysis inhibitors that selectively kill them. Thus, senescent VSMCs are unappreciated drivers of inflammation and matrix destruction in the vessel wall that do not require external stimuli or infiltrating cells.

The predominant view on the role of senescent VSMCs in atherosclerosis is that of inaction—failure to proliferate results in a VSMC-poor and matrix-poor unstable fibrous cap. Although no longitudinal study on the frequency of senescent VSMCs with plaque maturity exists, they are observed to be rare cells. Therefore, how few cells could affect plaque biology has been a puzzle. One possibility for this observed rarity is that senescent cells are cleared in vivo. However, without fragmentation, it is hard to rationalize how a phagocyte could engulf such a large cell. The data presented here indicate how small numbers of senescent VSMCs could have a much larger effect on plaque biology. First, once the SASP is established it is persistent and requires no external stimulation to maintain it. In vitro we have observed high-level secretion of cytokines/chemokines from senescent VSMCs for over 6 weeks, with no significant cell loss over many months (Humphry and Clarke, unpublished data, 2014, and Figure VII in the online-only Data Supplement). In contrast, macrophages rapidly undergo desensitization to activating stimuli, resulting in abatement of inflammatory responses. Therefore, a SASP can act as a constant source of inflammatory cytokines and chemokines for the life of the senescent VSMC within the plaque. Second, senescent VSMCs secrete IL-1α (the apical effector of the SASP) into the local milieu, as opposed to the typical cell surface expression on other senescent cell types,^9^ expanding the range of any effect. Normal VSMCs and ECs are responsive to IL-1, which results in the production of more proinflammatory cytokines and chemokines—effectively amplifying the signal. Indeed, control VSMCs treated with IL-1α, or media conditioned by senescent VSMCs, effectively phenocopy the SASP. Third, senescent VSMCs require no external stimulation or interaction with infiltrating cell types to generate vessel inflammation. Thus, small numbers of senescent VSMCs could theoretically seed inflammation throughout the vessel wall over extended time periods.

A key feature of atherogenesis is the constant recruitment of monocytes into the vessel wall and neointima. Several chemokines are components of the VSMC SASP, including MCP-1, MIP-1α/CCL3, and MIP-1β/CCL4. MCP-1 is well known to drive monocyte recruitment and deletion in mice results in dramatic reduction in recruitment and plaque growth.^24^ Indeed, we find MCP-1 to be the main SASP component that drives mononuclear cell migration (Figure 4A). Similarly, CCL3 and CCL4 are powerful chemokines for monocyte/macrophages and lymphocytes, and genetic blockade of CCL3/4 signaling decreases plaque size.^25,26^ However, CCL3 was originally identified as stem cell inhibitor, which inhibits proliferation of hemopoietic progenitors^27^ and other differentiated cells.^28,29^ Interestingly, CCR5^−/−^/ApoE^−/−^ mice (CCR5 is CCL3’s receptor) have smaller plaques with an unexplained 3-fold increase in VSMCs,^26^ whereas bone marrow CCR5^−/−^ does not affect plaque VSMC number.^25^ This suggests an inhibitory effect of CCR5 signaling on VSMC proliferation in vivo, and a potential cytostatic effect of the VSMC SASP. IL-6 and IL-8 are the principle SASP components and are found to be expressed by all senescent cell types.^8^ However, although IL-6 is powerfully associated with risk of MI,^30,31^ its precise contribution to atherogenesis is unclear and controversial.^32,33^ IL-8 is primarily a chemoattractant for neutrophils, but evidence suggests that it may promote firm adhesion of monocytes to ECs^34^ and cause their accumulation in plaques.^35^ In addition, IL-8 is an angiogenic factor that has been reported to induce angiogenesis in human coronary plaques,^36^ potentially driving growth of a pathological vasa vasorum. Together, the factors released by the VSMC SASP are highly likely to have a harmful impact on plaque growth and stability.

Identifying causation of in vitro findings in humans is hard. In addition, although atherosclerosis can be modeled in mice, VSMCs do not undergo senescence in these systems and establishment of SASPs in mouse cells is atypical.^37^ Human plaque VSMCs undergo telomere shortening-based senescence that is accelerated by DNA damage.^16^ Therefore, the lack of VSMC senescence in mice maybe because of their extremely long telomeres that delay senescence beyond the time frame of atherosclerosis experiments, and indeed mouse lifespan. Therefore, establishing direct consequences of VSMC senescence and the SASP in vivo is difficult. However, by examining SAβG-stained human carotid plaques, we have revealed senescent VSMC-like cells to express increased levels of the SASP-driver IL-1α. In addition, increased levels of IL-6 are found in SAβG-positive cells and adjacent normal cells, suggesting that the SASP can indeed activate other nearby cells to amplify inflammation. Finally, we find a significant relationship between SAβG cells and local accumulation of CD68-positive mononuclear cells, implying senescent cells can indeed drive chemotaxis in vivo.

Because of the multiplicity of risk factors, biological processes and cell types involved in atherosclerosis, therapeutic targeting to reduce growth or stabilize plaques is a challenging task. However, inflammation is well accepted to play a key role at all stages of atherogenesis from the early recruitment of monocytes that form fatty streaks through to final rupture. Because of this, and evidence for cholesterol crystal-activated inflammasomes producing IL-1β, the Canakinumab Anti-inflammatory Thrombosis Outcomes Study (CANTOS) was instigated. This study is the first to test whether blocking an inflammatory pathway in humans can reduce cardiovascular events. However, Canakinumab is a humanized monoclonal antibody specific for IL-1β, thus leaving the action of IL-1α completely unopposed. In many cases, IL-1β and IL-1α are cosecreted,^17,18^ whereas other atherosclerosis models show exclusive release of and dependence on IL-1α.^19^ Herein, we present data for another potentially important source of inflammation within the vessel wall that is also exclusively driven by IL-1α (Figure 2C–2F). Indeed, if a significant aspect of inflammation in atherosclerosis is driven by IL-1α, the effects in CANTOS may not seem as efficacious as required, or at worse the trial may fail losing a real therapeutic opportunity.

With every life lost to cardiovascular disease, the pressure to develop new treatment strategies and therapeutics builds. However, to achieve this, a detailed understanding of the precise mechanisms and processes underlying the disease is needed. We show that senescent VSMCs establish an inflammatory SASP driven by IL-1α that may have multiple negative effects on atherosclerosis. Although small numbers of senescent VSMCs are reported in plaques, secreted IL-1α can act on other normal vessel wall cells, inducing them to produce proinflammatory cytokines and to upregulate adhesion molecules, effectively amplifying the effect. We think senescent VSMCs may play an as yet unreported role in driving inflammation during atherosclerosis.

## Acknowledgments

We thank N. Figg for processing and sectioning of vessels.

## Sources of Funding

This study was supported by British Heart Foundation Grants FS/09/005/26845, FS/13/3/30038 (M.C.H. Clarke), and FS/10/034/28291 (M.R. Bennett).

## Disclosures

None.

## Supplementary Material

## Significance

Atherosclerotic plaques are the primary cause of ischemic heart disease and stroke—the leading causes of death. Plaque formation involves infiltration of myeloid cells into the vessel wall, proliferation, and migration of vascular smooth muscle cells (VSMCs) out into the forming plaque and interaction between these cell types. Inflammation is a key driver of the disease. However, overproliferation and DNA damage cause VSMCs to undergo senescence. Because VSMCs maintain plaques in an asymptomatic stable phenotype senescence is thought to contribute to the disease process by inaction. In contrast, we show that senescent VSMCs perpetually secrete multiple cytokines that drive inflammation and myeloid cell recruitment. This is driven by secreted interleukin-1α acting in an autocrine manner, which can also activate adjacent VSMCs and endothelial cells, amplifying the effect. This work contests the paradigm that senescent VSMCs are inactive bystanders in vascular disease, and that a potentially important source of inflammation requires blockade of interleukin-1α and not interleukin-1β.
