# Supplementary material for: Senescent Vascular Smooth Muscle Cells Drive Inflammation Through an Interleukin-1α–Dependent Senescence-Associated Secretory Phenotype
Source: Arterioscler Thromb Vasc Biol. 2015 Aug 26;35(9):1963–74. doi: 10.1161/ATVBAHA.115.305896 (PMC4548545; doi:10.1161/ATVBAHA.115.305896)
Supplement: Supplementary file 1 [file atv-35-1963-s001.pdf]

Materials and Methods – Online Only Data Supplement

All materials from Sigma-Aldrich unless otherwise stated.

Cell culture

Human aortic VSMCs were isolated from patients with local ethics committee approval. VSMCs and EL4 cells were cultured in DMEM, and THP-1 cells in RPMI 1640, all supplemented with 10 U/ml penicillin, 10 mg/ml streptomycin, 5 mg/ml L-glutamine and 10% FCS. HUVECS were cultured in basal media plus supplements (Lonza). VSMCs and HUVECs were cultured to ~80% confluence before passaging. EL-4 and THP-1 cells were maintained at 4-10x10^5 cells/ml. Cells were treated where indicated with; IL-1α pAb (2 μg/ml), IL-1α (10ng/ml) (all PeproTech), IL-1β pAb (2 μg/ml; R&D), LPS (1 μg/ml). To induce senescence VSMCs were treated with bleomycin sulphate (50-200 μg/ml; 3h; Enzo) before incubation (typically 11-14 d). For replicative senescence, VSMCs were passaged until growth arrest (typically p = 14-18). To analyse the SASP control and senescent VSMC were adhered overnight, washed, and incubated in serum replacement media (#1; Sigma) for 24 h. Media was refreshed and conditioned media collected after 6 h, clarified and cytokines assayed by ELISA (IL-6, IL-8, MCP-1; PeproTech), Cytomix (IL-2, IL-1α; eBioscience), Cytometric bead array (BD) or an antibody array (Ray Biosciences) according to manufacturers instructions. To detect IL-1α-specific activity control VSMCs were adhere overnight and then incubated in serum free (S/F) media for ~24 h, whilst HUVECS were only adhered overnight. Fresh media was added along with treatments as indicated and incubated for 6 h. EL4 cells were washed and plated in S/F media along with treatments and incubated for 24 h. Conditioned media was collected, clarified, and cytokines assayed as above. Specific IL-1α activity was inferred with a neutralising antibody. To make necrotic lysates, cells in S/F DMEM were disrupted by three freeze-thaw cycles in liquid N2, clarified and stored at -80°C. To assess cell viability in long-term culture, senescent cells were plated into flasks and allowed to adhere for 1 w. Flasks were marked on the bottom and repeatedly imaged at the same field of view every 3-4 days, before manual enumeration.

Cell and tissue staining

For SABG staining cells were fixed in formaldehyde (2%; 3 mins), washed, and incubated at 37°C in staining solution (40 mM citric acid/sodium phosphate pH 6.0; 5 mM $K_4[Fe(CN)_6]3H_2O$; 5 mM $K_3[Fe(CN)_6]$; 150 mM NaCl; 2 mM $MgCl_2$) with fresh X-Gal added (1 mg/ml). To assess proliferation VSMCs were incubated with BrdU (10μM; 24h), fixed with formaldehyde (2%; 15mins), treated with HCl (2M; 30min), permeabilised in Np-40 (0.5%; 2 mins), blocked with goat serum (5%; 1 h), and incubated with anti-BrdU antibody (1:500; 4°C; 16h; AbCam). Washed cells were incubated with biotinylated 2ry antibody (1:500; 1), then ABComplex (30 mins), before visualisation with DAB (all Vector). To assess DNA damage VSMCs were fixed with methanol (10 mins; -20°C), blocked in SignalStain (1h), incubated in anti-pATM or pH2AX antibody (1:100; 4°C; 16h; all Cell Signaling Technology). αSMA was visualised after fixation with formaldehyde (2%; 15mins), permeabilised in Np-40 (0.5%; 2 mins), blocking, and incubated with anti-αSMA (1:500; 4°C; 16h; Dako). Washed cells were incubated AlexaFluor568 or 488 (1:500; 1 h), before mounting in Prolong Gold ± DAPI (both Invitrogen). Whole carotid plaques were lightly fixed in formaldehyde (2%) before SAβG staining as above. After processing paraffin sections were cleared before antigen retrieval with sodium citrate (10mM; pH 6), blocking in H2O2 (3%; 10 mins) and then horse serum (5%; 1 h), and incubated with antibodies to anti-α smooth muscle actin (1:500; 4°C; 16 h; Dako); anti-IL-1α (1:40; 4°C; 16 h; Abcam); anti-IL-6 (1:5000; 4°C; 16 h; ProteinTech); anti-CD68 (1: 500; 4°C; 16 h; Dako) before visualisation with DAB, as above. Cell death was assessed with propidium iodide and Hoechst 33342 staining (both 1μg/ml). Imaging was performed on a BX51 or IX71 (Olympus) using CellD or OpenLab software. For labelling of membrane IL-1α (1:40; R&D), VCAM-1, ICAM-1, or E-selectin (all 1:20; Biolegend) cells were stained in BSA (1%)/sodium azide (0.05%) FACS buffer (45 mins; RT), washed and analysed on a C6 flow cytometer (Accuri BD).

LDH assay
LDH activity was determined by its enzymatic oxidation of NADH. NADH (0.2mM), sodium pyruvate (1.6mM) and test sample were mixed in the volumetric ratio of 21:4:2 in Tris/NaCl buffer and monitored kinetically in a spectrophotometer at 339nm (37˚C). Cell lysates made by freeze-thaw were used as a positive control.

Transmigration assay
Control or senescent VSMCs were plated (24 well; $15x10^3$), incubated overnight, washed and incubated in serum replacement media (#1) for 24 h ± IL-1α pAb (2 μg/ml; Peprotech). Transwell inserts (8μm; Falcon) were added, along with MCP-1 (6μg/ml; Biolegend) or IL-8 (2μg/ml; Abcam) neutralising antibodies to the bottom, where indicated. $1x10^5$ green labelled (CMFDA; Invitrogen) THP-1 cells were added to the top of each transwell and the plate incubated (4 h). Transwells were removed and the lower media collected along with gentle washing of the underside. Green THP-1 cells/ml were enumerated by flow cytometry (Accuri C6).

In vivo recruitment
Experiments were conducted under UK Home Office licensing. Male littermate C57BL/6J mice of the same weight were injected intraperitoneally with $1x10^5$ control or senescent live VSMCs. After 6 h the peritoneal cavity was lavaged with 5 ml of PBS. Neutrophils, monocyte/macrophages and lymphocytes were enumerated by FACS following staining for GR-1 (2.5μg/ml; Biolegend) and F4/80 (1:10; ABD Serotec), and FSC/SSC gating.

Gelatin zymography
Control, IL-1α-primed and senescent VSMCs were incubated in DMEM with serum replacement (#1; 24 h). Clarified supernatant was concentrated 20-fold before addition of non-reducing Laemmli buffer, electrophoresed on a gelatin zymography gel (Novex), re-natured in 2.5% Triton X100 (2.5%; 30 mins) before incubation in zymography developing buffer (16 h; Novex), and Coomassie staining.

Collagen Assay
Cells were plated, incubated in 1% serum and conditioned media collected after 3 d. Proteins were concentrated by overnight PEG precipitation and collagen content analysed using the Sircol assay (Biocolor). Values were normalised to cell number.

Cell number assessment
VSMCs were fixed with formaldehyde (2%; 15 mins), washed and stained with Hoechst 33342 (10μg/ml; 10 mins), before measurement at 360/460 nm. For crystal violet, fixed cells were incubated in methanol/acetic acid (3:1; 5 mins), washed (H_2O) thoroughly, dryed, stained with crystal violet (0.1%; PBS; 10 mins), washed and dried. Retained dye was solubilised with acetic acid (10%) before measurement at 590 nm.

Quantitative PCR
RNA was isolated using trizol reagent and DNAse treated (Ambion) before reverse transcription with mMLV and oligo(dT) (Promega). TaqMan probe/primers were used with AmpliTaq gold (Life Technologies) in a RotorGene thermocycler (Corbett). Analysis utilised standard curves with specific expression level normalised to GUSB and TBP.

Cellular respiration analysis
Respiration was measuring using a Seahorse XF96 extracellular flux analyser according to the manufacturer's instructions. Briefly, control and senescent VSMCs were plated into XF96 microplates and allowed to settle for 48 hours. A XF96 fluxpak was calibrated overnight and then the injection ports filled to inject oligomycin (1μg/ml), FCCP (1μM), 2-DG (10mM) and

antimycin A/rotenone (10/1µM). Standard analysis of OCR and ECAR was performed and values normalised to cell number by crystal violet staining.

XBP1 splicing
Splicing of XBP1 was assessed using RT-PCR (Fwd: CCTGGTTGCTGAAGAGGAGG; Rev: CCATGGGGAGATGTTCTGGAG) that amplified at 145bp for unspliced and 119bp for spliced, and qPCR using Sybr Green (Fwd: AAACAGAGTAGCAGCGCAGACTGC; Rev: TCCTTCTGGGTAGACCTCTGGGAG).

Statistics
Data are presented as mean ± SEM, unless otherwise stated. All assays that produced continuous data, with the exception of flow cytometry and mouse experiments, were performed in duplicate. n = an individual experimental replicate. Parametric tests were employed for analysis of continuous data, conducted using a one-way, two-tailed ANOVA (Excel). Significance is as stated, but always $p = <0.05$. Non-significant (NS) data was considered anything $p = >0.05$.
