# Supplementary material for: Senescent Vascular Smooth Muscle Cells Drive Inflammation Through an Interleukin-1α–Dependent Senescence-Associated Secretory Phenotype
Source: Arterioscler Thromb Vasc Biol. 2015 Aug 26;35(9):1963–74. doi: 10.1161/ATVBAHA.115.305896 (PMC4548545; doi:10.1161/ATVBAHA.115.305896)
Supplement: Supplementary file 2 [file atv-35-1963-s002.pdf]

I

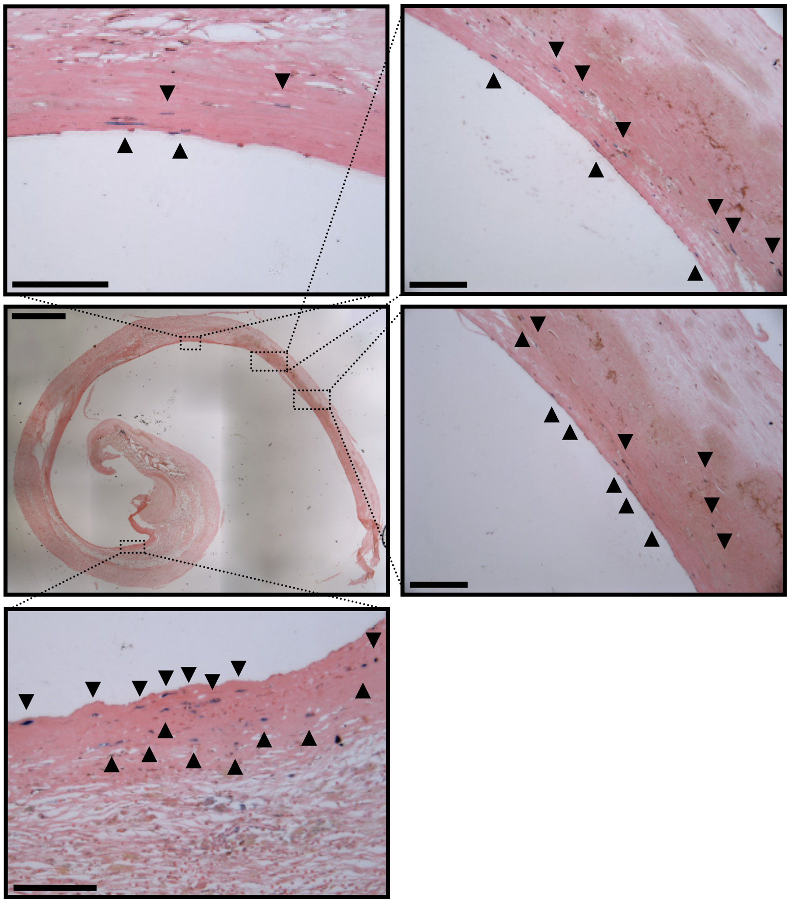

Figure I: Images from a whole human carotid endartarectomy stained for SABG showing senescent cells in the fibrous cap of the mature plaque, but also in more peripheral regions overlying areas of intimal thickening. Scale bars represent 100μm (high power) and 1mm (low power)

II

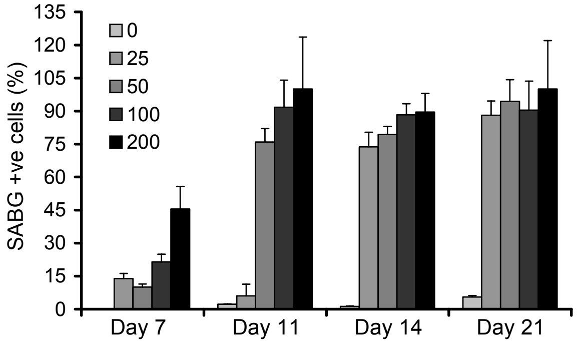

III

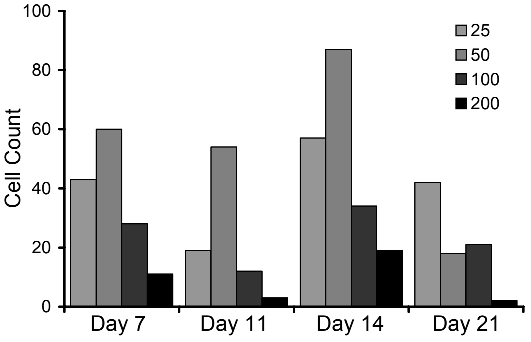

IV

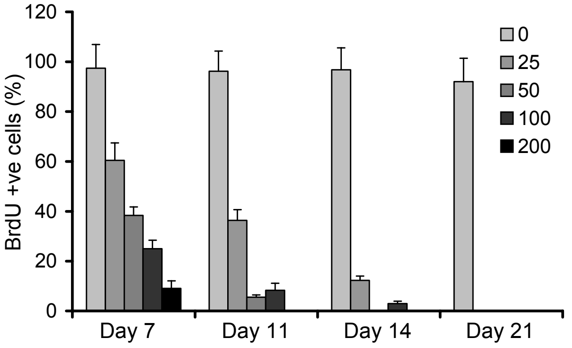

V

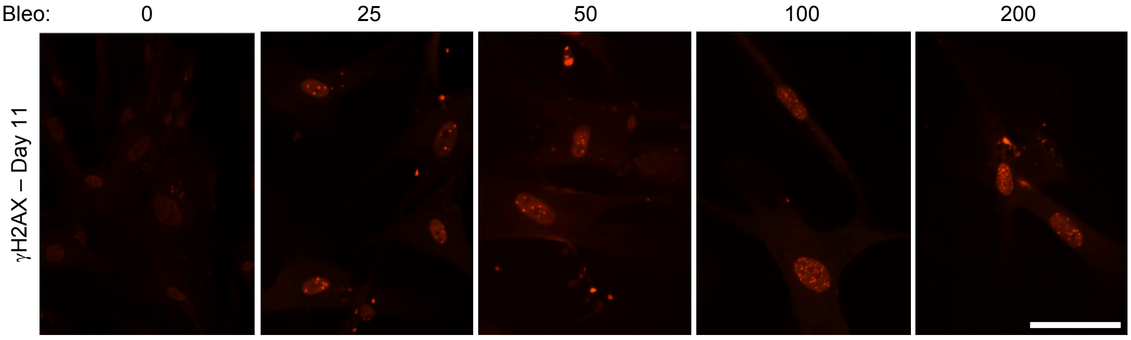

Figure II-IV: VSMCs were treated with Bleomycin at the concentrations indicated (µg/ml) and harvested at the time indicated before enumeration of SABG +ve cells **(II)**, relative number of cells **(III)** or BrdU +ve cells **(IV)**. Data represent mean ± SEM of n = ≥3

Figure V: VSMCs were treated with Bleomycin at the concentrations indicated (µg/ml) and harvested at day 11 before staining for persistent unrepaired DNA with γH2AX. Scale bar represents 50µm.

VI

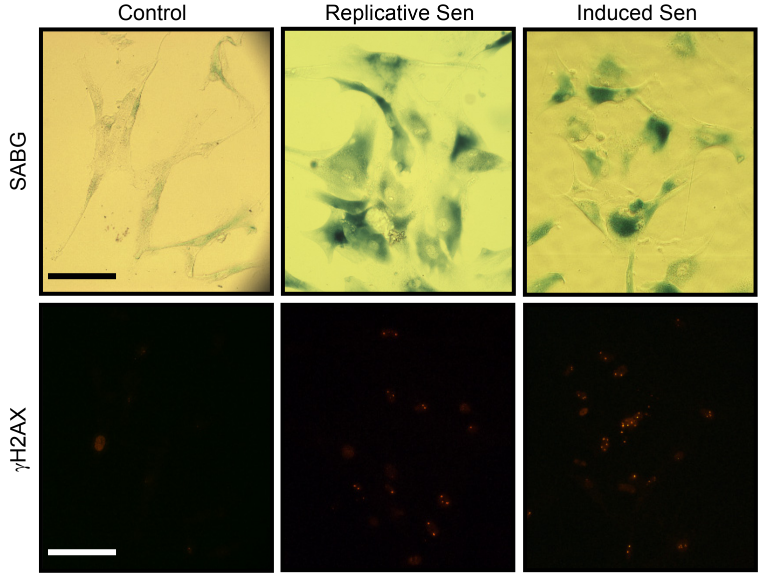

VII

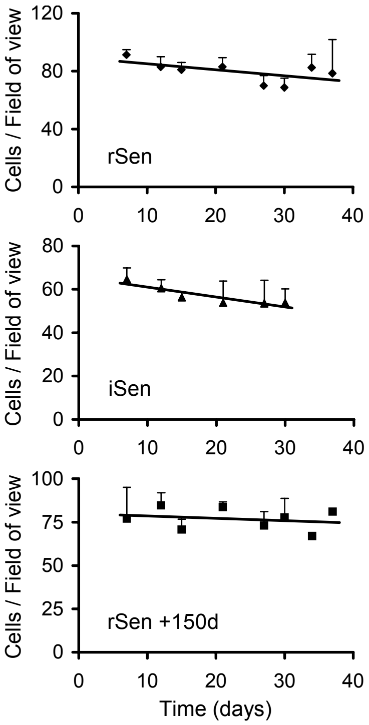

VIII

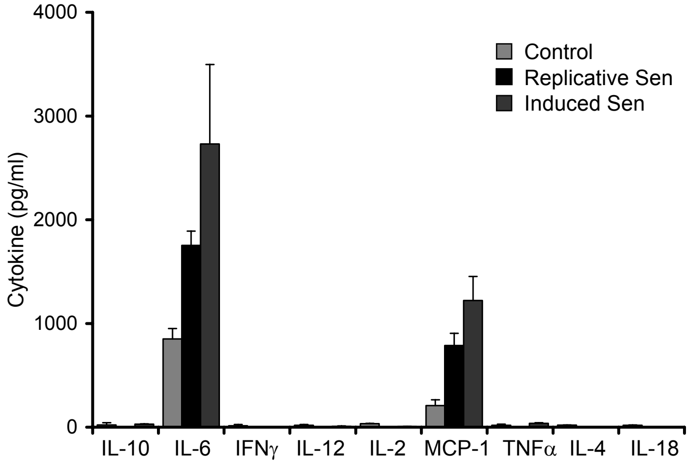

IX

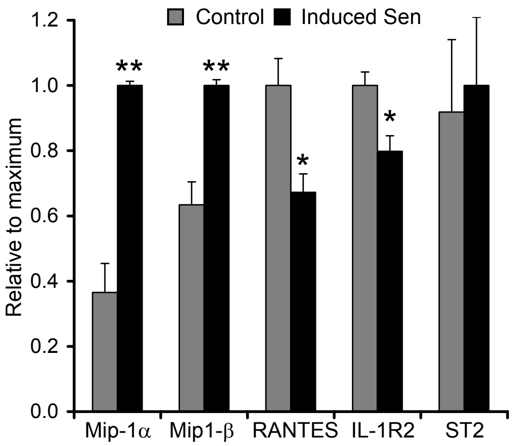

Figure VI: Replicative or induced senescent VSMCs were stained for SABG and γH2AX. Scale bars represent 50μm.

Figure VII: Replicative or induced senescent VSMCs were cultured in flasks for 7 d or 150 d, as indicated, before repeated imaging and enumeration of cells per field of view. Data represent mean ± SD of 3 fields of view per flask.

Figure VIII, IX: Cytokine and chemokine content of conditioned media from control and senescent VSMCs measured by cytometric bead array **(VIII)**, or antibody array **(IX)**.

Supplemental Figures

X

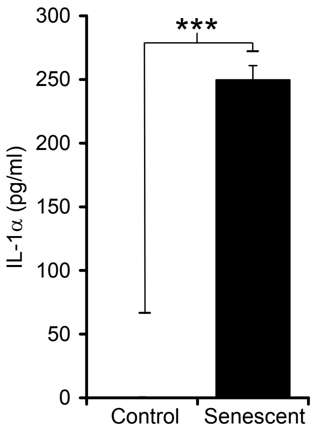

XI

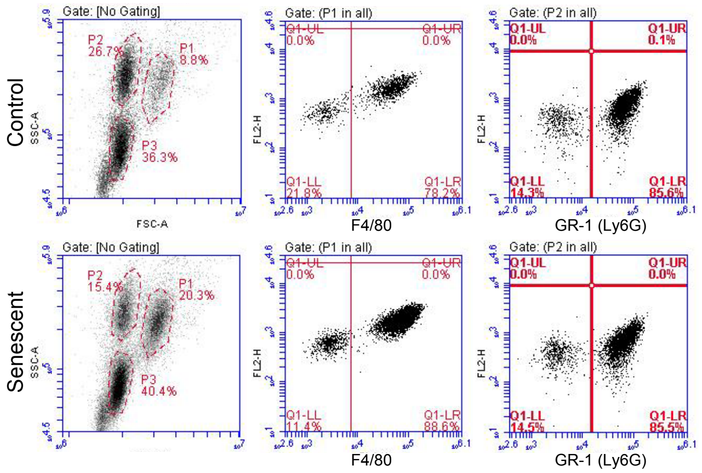

XII

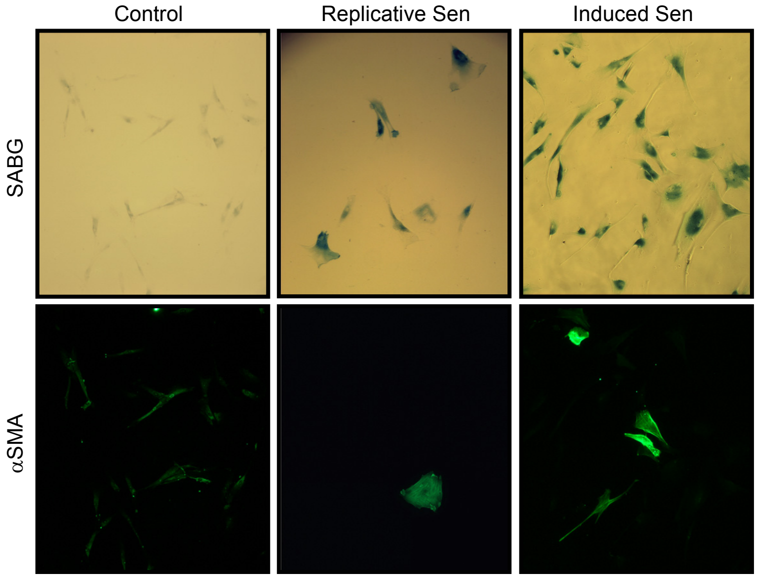

Figure X: IL-1α content of conditioned media from control and senescent VSMCs measured by ELISA. Data represent mean ± SD of n = 3; p = ***≤0.005.

Figure XI: Example flow cytometry plots of cells lavaged from the peritoneum. F4/80 positive monocyte/macrophages reside in gate 1; GR-1 (Ly6G) positive neutrophils reside in gate 2; whilst lymphocytes, clearly separated by FSC/SSC, reside in gate 3.

Figure XII: Replicative or induced senescent VSMCs were stained for SABG and αSmooth muscle actin (αSMA). Control cells show an even expression, whilst senescent cells show polarization between low and very high expression. Scale bars represent 50μm.

XIII

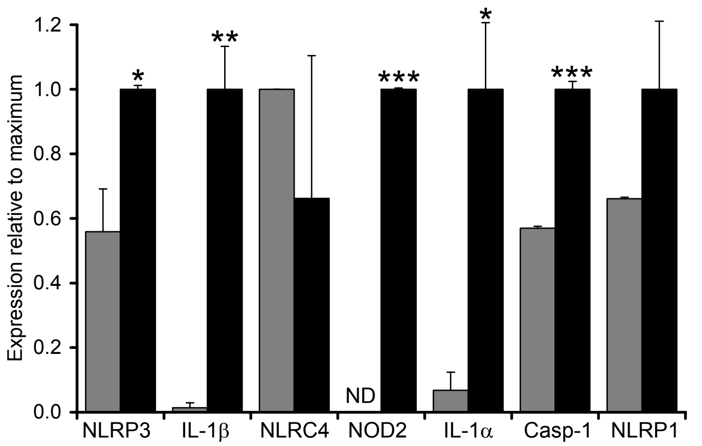

XIV

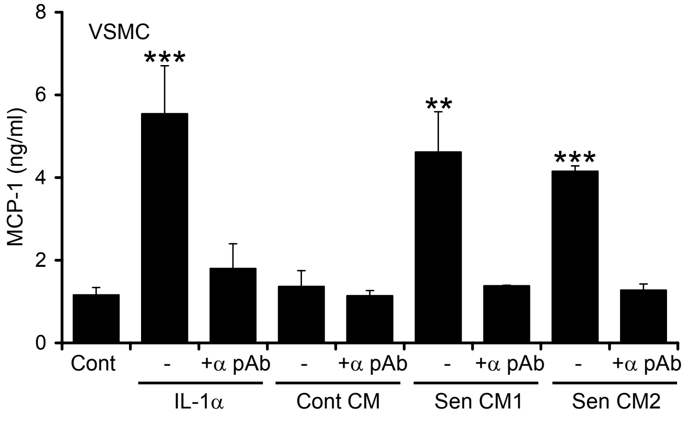

XV

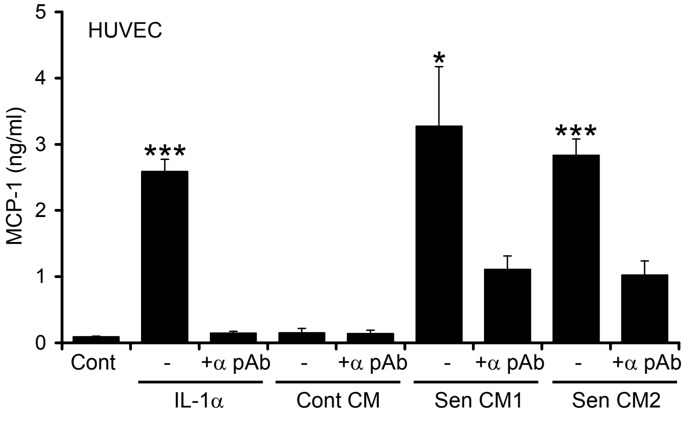

Figure XIII: Relative expression of transcripts for inflammasome associated components in control (grey) or IL-1α-treated (black) VSMCs. Data represent mean ± SEM of n = 2; p = *≤0.05, **≤0.02, ***≤0.005.

Figure XIV, XV: MCP-1 content of cell lysates from control VSMCs **(XIV)** or HUVECs **(XV)** incubated with IL-1α or conditioned media (CM) from control or two senescent VSMC cultures, ± neutralising IL-1α pAb. Data represent mean ± SD of n = 3 (XIV), 2 (XV); p = *≤0.05, **≤0.02, ***≤0.005.
